# Functional variants of the *chitinase 3‐like 1* gene are associated with clinicopathologic outcomes and progression of prostate cancer

**DOI:** 10.1111/jcmm.18012

**Published:** 2023-10-30

**Authors:** Yu‐Ching Wen, Chia‐Yen Lin, Kuo‐Hao Ho, Yung‐Wei Lin, Chi‐Hao Hsiao, Shian‐Shiang Wang, Lun‐Ching Chang, Shun‐Fa Yang, Ming‐Hsien Chien

**Affiliations:** ^1^ Department of Urology, School of Medicine, College of Medicine and TMU Research Center of Urology and Kidney (TMU‐RCUK) Taipei Medical University Taipei Taiwan; ^2^ Department of Urology, Wan Fang Hospital Taipei Medical University Taipei Taiwan; ^3^ Division of Urology, Department of Surgery Taichung Veterans General Hospital Taichung Taiwan; ^4^ School of Medicine Chung Shan Medical University Taichung Taiwan; ^5^ School of Medicine National Yang Ming Chiao Tung University Taipei Taiwan; ^6^ Graduate Institute of Clinical Medicine, College of Medicine Taipei Medical University Taipei Taiwan; ^7^ International Master/PhD Program in Medicine, College of Medicine Taipei Medical University Taipei Taiwan; ^8^ Department of Applied Chemistry National Chi Nan University Nantou Taiwan; ^9^ Department of Mathematical Sciences Florida Atlantic University Boca Raton Florida USA; ^10^ Institute of Medicine Chung Shan Medical University Taichung Taiwan; ^11^ Department of Medical Research Chung Shan Medical University Hospital Taichung Taiwan; ^12^ Pulmonary Research Center, Wan Fang Hospital Taipei Medical University Taipei Taiwan; ^13^ Traditional Herbal Medicine Research Center Taipei Medical University Hospital Taipei Taiwan; ^14^ TMU Research Center of Cancer Translational Medicine Taipei Medical University Taipei Taiwan

**Keywords:** cancer incidence, chitinase 3‐like 1, clinicopathologic progression, prostate cancer, single‐nucleotide polymorphism

## Abstract

Chitinase 3‐like 1 (CHI3L1 or YKL40) is a secreted glycoprotein highly expressed in advanced stages of several cancer types, including prostate cancer (PCa). Impacts of genetic variants of *CHI3L1* on PCa development have not yet been investigated. The most common well‐studied genetic variations are single‐nucleotide polymorphisms (SNPs). Therefore, the objective of this study was to explore associations of *CHI3L1* SNPs with both the susceptibility to PCa and its clinicopathological development. Three promoter SNPs, rs6691378 (−1371, G>A), rs10399805 (−247, G>A) and rs4950928 (−131, C>G), and one non‐synonymous SNP, rs880633 (+2950, T>C), were analysed using a TaqMan allelic discrimination assay for genotyping in a cohort of 701 PCa patients and 701 healthy controls. Results indicated that there were no significant associations of PCa susceptibility with these four *CHI3L1* SNPs. However, among elderly PCa patients (aged >65 years), it was observed that polymorphic variants (GA + AA) of *CHI3L1* rs6691378 and 10399805 were significantly linked to reduced risks of several clinicopathological characteristics, including a high Gleason grade, advanced pathologic T stage and tumour cell invasion. Moreover, analyses of The Cancer Genome Atlas database revealed that *CHI3L1* expression levels were elevated in PCa tissues compared with normal tissues. Interestingly, higher *CHI3L1* expression levels were found to be associated with longer progression‐free survival rates in PCa patients. Our findings indicated that levels of CHI3L1 may influence the progression of PCa, and the rs6691378 and 10399805 SNP genetic variants of *CHI3L1* are linked to the clinicopathological development of PCa within a Taiwanese population.

## 1 INTRODUCTION

Every year, over 34,700 men succumb to prostate cancer (PCa) in the United States, positioning it as the second leading contributor to cancer‐related fatalities among males on a global scale.^1^ While early‐stage PCa can be effectively managed through a radical prostatectomy (RP) and radiotherapy, the same cannot be said for metastatic PCa. Although androgen‐deprivation therapy (ADT) is initially effective in the majority of patients with advanced and metastatic PCa, there is an unfortunate trend where a significant number of patients eventually experience biochemical recurrence.^2^ Several prognostic factors were reported to predict the outcome of primary treatment and prognosis for PCa, including prostate‐specific antigen (PSA) levels, Gleason grade and score, and the stage of the disease.^3^, ^4^, ^5^ Nonetheless, conventional clinicopathological parameters often fall short in accurately predicting prognoses. For instance, elevated serum PSA levels can be observed in cases of PCa and also in benign conditions like prostatic hyperplasia and prostatitis.^6^ The PSA test suffers from both limited sensitivity and specificity. Furthermore, the significance of PSA declines in the later stages of the disease.^7^ Hence, given the imperative of an early PCa diagnosis to prevent metastasis and facilitate timely treatment, it is crucial to conduct research aimed at identifying novel and efficient predictive biomarkers.

The chitinase‐3‐like‐1 (CHI3L1) protein, also known as YKL40, is categorized as a secretory glycoprotein and is a member of glycoside hydrolase family 18 (GH18) of chitinases. CHI3L1 is overexpressed in many human cancer types such as lung, liver, breast, colorectal, ovary and cervical cancers. Moreover, a higher serum CHI3L1 level has shown promise as a valuable prognostic biomarker in different cancers.^8^, ^9^, ^10^, ^11^, ^12^, ^13^ In PCa, elevated serum CHI3L1 levels were also documented in individuals with primary PCa compared with those with benign prostate hyperplasia (BPH), suggesting a potential role for CHI3L1 in influencing the development of PCa.^14^ Additionally, higher serum CHI3L1 levels were linked to shorter overall survival (OS) and early mortality among metastatic PCa patients undergoing ADT^15^ and docetaxel chemotherapy.^16^ Most importantly, there is a proposition that CHI3L1, compared with the widely used PSA, could provide more informative insights into predicting tumour burdens and the potential for metastasis.^17^

The human *CHI3L1* gene is located on chromosome 1q31–1q32, and several variants of the *CHI3L1* gene were identified. For example, single‐nucleotide polymorphisms (SNPs) located in promoter (rs4950928, rs10399931 and rs10399805), exon (rs880633) and intron (rs2071579, rs1538372, rs2275353 and rs946259) regions of the *CHI3L1* gene were identified as being associated with its serum levels within the general population. These associations were observed at or below significant genome‐wide association levels.^18^ Therefore, the *CHI3L1* rs4950928 C allele was reported to be associated with risk of rectal cancer and increased serum levels of CHI3L1 in Egyptians.^19^ Moreover, the *CHI3L1* rs880633 C allele was also correlated with the risk of liver cancer and higher serum CHI3L1 levels compared with the rs880633 T allele.^20^ Furthermore, Su et al. indicated a significant association of the homozygous phenotype (AA) for the minor allele of *CHI3L1* rs10399805 and rs6691378 with a lower risk of developing lymph node (LN) metastasis in Taiwanese with oral cancer.^21^ Despite several studies having investigated the clinical significance and functional role of CHI3L1 in PCa, the effects of *CHI3L1* genetic variants on PCa have not been explored. In this study, our aim was to examine associations of SNPs within the *CHI3L1* gene with the risk of the clinicopathological development of PCa in a Taiwanese population.

## 2 MATERIALS AND METHODS

### 2.1 Healthy volunteers and patients with PCa

This retrospective study involved two cohorts: a group of 701 Taiwanese PCa patients and a matched set of 701 healthy male controls with the same ethnic background and residing in a similar geographic area. PCa diagnoses were histologically confirmed in all cases following a robotic‐assisted laparoscopic RP at Taichung Veterans General Hospital (Taichung, Taiwan) between 2012 and 2018. Demographic characteristics and medical details of patients were collected from their medical records at the time of the PCa diagnosis. These details included PSA values, pathologic Gleason grade, pathologic T (tumour) and N (node) staging, invasion areas of tumour cells (such as seminal vesicle, perineural and lymphovascular involvement) and the D'Amico classification. Prior to blood sampling, informed consent was obtained from each participant, and the study was approved by the ethics committee of Taichung Veterans General Hospital (ethics approval no. CE19062A).

### 2.2 Blood sample collection and genomic DNA extraction

Peripheral blood of recruited subjects was aseptically collected through venipuncture and preserved in tubes containing EDTA for the purpose of DNA purification. DNA was extracted using a QIAamp DNA Blood Mini Kit from Qiagen (Valencia, CA, USA). Extracted DNA was dissolved in Tris‐EDTA (TE) buffer, consisting of 10 mM Tris and 1 mM EDTA at pH 7.8. Subsequently, the DNA purity was assessed using a Nanodrop‐2000 spectrophotometer (Thermo Fisher Scientific, Waltham, MA, USA) to determine the ratio of absorbances at 260 and 280 nm. The final DNA preparations were then stored at a temperature of −20°C in preparation for a subsequent real‐time polymerase chain reaction (PCR) analysis.

### 2.3 Selection and determination of *CHI3L1* SNPs

In total, four *CHI3L1* genetic variants were selected for analysis. These included three promoter SNPs of rs6691378 (−1371, G/A), rs10399805 (−247, G/A) and rs4950928 (−131, C/G), and one non‐synonymous SNP of rs880633 (2950, T/C). These specific SNPs were chosen based on information from the Chinese HapMap dataset, which is focused on the Han Chinese population in Beijing, China. These four SNPs were previously implicated in impacting various aspects related to cancer. They were associated with influencing the occurrence, severity, or progression of different cancer types. Additionally, these genetic variants were linked to modulation of the expression of the *CHI3L1* gene.^19^, ^20^, ^21^, ^22^ The allelic discrimination of these four *CHI3L1* SNPs including rs4950928 (assay ID: C_27832042_10), rs6691378 (assay ID: C_29933614_10), rs880633 (assay ID: C_11891591_1) and rs10399805 (assay ID: C_29969647_10) was evaluated by utilizing the TaqMan SNP Genotyping Assay. This analysis was conducted using the ABI StepOnePlus™ Real‐Time PCR System, a product of Thermo Fisher Scientific. This approach allows the differentiation of allelic variants at these specific positions within the *CHI3L1* gene, facilitating the genotyping of these SNPs. Detailed processes regarding DNA genotyping were published in our previous study.^23^

### 2.4 Bioinformatics analysis

The UCSC Xena database (https://xena.ucsc.edu/) facilitated access to clinical data and messenger (m)RNA sequencing information from prostate adenocarcinoma (PRAD) samples sourced from The Cancer Genome Atlas (TCGA). Within this context, we conducted a comparative analysis of *CHI3L1* gene expression levels across a range of clinical features, including Gleason scores, clinical stages, pathological tumour sizes, lymph node statuses and the presence of distal metastases. For two‐group comparisons, we employed the Wilcoxon signed‐rank test, while clinical features with more than two groups were subjected to the Kruskal–Wallis test, followed by post hoc Dunn's tests. To explore the association between CHI3L1 and patients' progression‐free survival (PFS), we applied a log‐rank test, and high and low CHI3L1 expression groups were determined based on the median cut‐off point of CHI3L1.

### 2.5 Statistical analysis

To assess the association between CHI3L1 genotypic frequencies and clinicopathologic features, we employed multivariate logistic regression models. These models were utilized to calculate odds ratios (ORs), adjusted ORs (AORs) and corresponding 95% confidence intervals (CIs). All statistical analyses were conducted with the SAS software program (vers. 9.1, 2005; SAS Institute, Cary, NC, USA). The threshold for statistical significance was set to *p* < 0.05.

## 3 RESULTS

### 3.1 Demographic characteristics of recruited PCa patients

Demographic and clinicopathological characteristics of 701 PCa patients who received an RP are shown in Table 1. Our study cohort predominantly consisted of older individuals, with 57.8% being over the age of 65 years. Our recruited cohort was consistent with previous reports which indicated that nearly 60% of PCa cases are diagnosed in patients over the age of 65 years.^24^ The majority of patients exhibited early‐stage tumours (clinical T1 or T2 stage, 86%), falling within Gleason grade groups 1 or 2 (60.1%) and presenting with perineural invasion (73.5%). Lymph node metastasis (N0) was absent in 91.4% of cases, as were lymphovascular invasion (84.2%) and seminal vesicle invasion (78.7%). Categorizing patients according to the D'Amico risk classification, over half of the PCa patients (50.4%) were classified as having a high risk (>50% likelihood) of experiencing recurrence within 5 years following treatment.

**TABLE 1:** Distributions of demographical characteristics of 701 patients with prostate cancer.

Variable	Patients (*N* = 701)
Age at diagnosis (years)	
≤65	296 (42.2%)
>65	405 (57.8%)
PSA at diagnosis (ng/mL)	
≤10	333 (47.5%)
>10	368 (52.5%)
Pathologic Gleason grade group	
1 + 2	421 (60.1%)
3 + 4 + 5	280 (39.9%)
Clinical T stage	
1 + 2	603 (86.0%)
3 + 4	98 (14.0%)
Pathologic T stage	
2	371 (52.9%)
3 + 4	330 (47.1%)
Pathologic N stage	
N0	641 (91.4%)
N1	60 (8.6%)
Seminal vesicle invasion	
No	552 (78.7%)
Yes	149 (21.3%)
Perineural invasion	
No	186 (26.5%)
Yes	515 (73.5%)
Lymphovascular invasion	
No	590 (84.2%)
Yes	111 (15.8%)
Biochemical recurrence	
No	479 (68.3%)
Yes	222 (31.7%)
D'Amico classification	
Low risk/ Intermediate risk	348 (49.6%)
High risk	353 (50.4%)

Abbreviation: PSA, prostate‐specific antigen.

### 3.2 Impacts of *CHI3L1* genetic polymorphisms on the PCa incidence

We then proceeded to investigate potential correlations between the four selected SNPs (rs4950928 [C/G], rs6691378 [G/A], rs880633 [T/C] and rs10399805 [G/A]) within the *CHI3L1* gene and PCa occurrence. We initially examined genotype frequencies of these SNPs across the entire recruited population. As illustrated in Table 2, predominant distribution frequencies of *CHI3L1* rs4950928, rs6691378, rs880633 and rs10399805 SNPs in PCa patients included homozygous C/C, G/G and G/G for rs4950928, rs6691378 and rs10399805 loci, respectively, and heterozygous T/C for the rs880633 locus. Genotypic distributions of these four *CHI3L1* SNPs in the control group conformed to Hardy–Weinberg equilibrium (*χ* ^2^ value = 0.089, *p* = 0.765 for rs4950928 C>G; *χ* ^2^ value = 0.358, *p* = 0.549 for rs6691378 G>A; *χ* ^2^ value = 0.061, *p* = 0.805 for rs880633 T>C, and *χ* ^2^ value = 2.048, *p* = 0.152 for rs10399805 G>A). Employing AORs with 95% CIs calculated through multiple logistic regression models with age as a covariate, we examined associations between *CHI3L1* SNPs and PCa incidence. Notably, in the context of the recruited Taiwanese population, our analyses revealed no significant links between *CHI3L1* SNPs and PCa occurrence. This outcome held true whether assessed through a dominant or codominant model, as detailed in Table 2.

**TABLE 2:** Adjusted odds ratios (AORs) and 95% confidence intervals (CIs) of prostate cancer associated with *CHI3L1* genotypic frequencies.

Genotype	Controls (*N* = 701) (%)	Patients (*N* = 701) (%)	AOR (95% CI)	*p‐*value
rs4950928				
CC	510 (72.8%)	530 (75.6%)	1.000 (reference)	
CG	177 (25.2%)	156 (22.3%)	1.031 (0.778–1.366)	0.833
GG	14 (2.0%)	15 (2.1%)	1.378 (0.613–3.096)	0.438
CG + GG	191 (27.2%)	171 (24.4%)	1.057 (0.805–1.388)	0.691
rs6691378				
GG	315 (44.9%)	316 (45.1%)	1.000 (reference)	
GA	315 (44.9%)	314 (44.8%)	0.954 (0.743–1.224)	0.711
AA	71 (10.1%)	71 (10.1%)	0.858 (0.563–1.306)	0.474
GA + AA	386 (55.1%)	385 (54.9%)	0.936 (0.738–1.188)	0.588
rs880633				
TT	305 (43.5%)	305 (43.5%)	1.000 (reference)	
TC	317 (45.2%)	311 (44.4%)	0.921 (0.715–1.186)	0.523
CC	79 (11.3%)	85 (12.1%)	0.987 (0.669–1.456)	0.946
TC + CC	396 (56.5%)	396 (56.5%)	0.934 (0.735–1.187)	0.578
rs10399805				
GG	331 (47.2%)	337 (48.1%)	1.000 (reference)	
GA	313 (44.7%)	300 (42.8%)	0.903 (0.705–1.158)	0.422
AA	57 (8.1%)	64 (9.1%)	0.916 (0.585–1.435)	0.701
GA + AA	370 (52.8%)	364 (51.9%)	0.905 (0.714–1.148)	0.412

*Note*: AORs with their 95% CIs were estimated by multiple logistic regression models after controlling for age.

### 3.3 Impacts of *CHI3L1* genetic polymorphisms on clinicopathologic features of PCa patients

Subsequently, we examined potential correlations between *CHI3L1* genetic polymorphisms and various clinicopathological features among all PCa patients. The patient cohort was divided into two subgroups: individuals possessing homozygous wild‐type (WT) alleles and those carrying at least one polymorphic allele. However, our analysis revealed no significant associations between *CHI3L1* rs4950928, rs6691378, rs880633 and rs10399805 SNPs with clinicopathological features (Tables 3 and 4). We extended our investigation to explore associations between clinicopathological characteristics and the four *CHI3L1* SNPs specifically within the elderly PCa patient subgroup (aged >65 years). Our findings indicated that individuals carrying at least one minor allele of rs6691378 (GA and AA) exhibited a notably reduced risk of developing a high Gleason grade (3–5) (OR = 0.656, 95% CI: 0.441–0.977, *p* = 0.038), advanced pathologic T stage (T3 or T4) (OR = 0.582, 95% CI: 0.391–0.865, *p* = 0.007) and seminal vesicle invasion (OR = 0.508, 95% CI: 0.320–0.806, *p* = 0.004), as depicted in Table 5. Furthermore, *CHI3L1* rs10399805 polymorphisms also displayed significant associations with the pathologic Gleason grade (OR = 0.637, 95% CI: 0.429–0.947, *p* = 0.026), T stage (OR = 0.564, 95% CI: 0.380–0.837, *p* = 0.004), seminal vesicle invasion (OR = 0.597, 95% CI: 0.377–0.946, *p* = 0.027) and perineural invasion (OR = 0.608, 95% CI: 0.381–0.970, *p* = 0.036) among elderly PCa patients carrying at least one polymorphic A allele (Table 6). These data provide valuable insights into potential associations between *CHI3L1* genetic variations and specific clinicopathological characteristics in the context of older PCa patients.

**TABLE 3:** Odds ratios (ORs) and 95% confidence intervals (CIs) of the clinical status and *CHI3L1* rs4950928 and rs6691378 genotypic frequencies in 701 patients with prostate cancer.

Variable	rs4950928				rs6691378			
	CC (*N* = 530)	CG + GG (*N* = 171)	OR (95% CI)	*p‐*value	GG (*N* = 316)	GA + AA (*N* = 385)	OR (95% CI)	*p‐*value
PSA at diagnosis (ng/mL)								
≤10	248 (46.8%)	85 (49.7%)	1.00	0.507	146 (46.2%)	187 (48.6%)	1.00	0.532
>10	282 (53.2%)	86 (50.3%)	0.890 (0.630–1.256)		170 (53.8%)	198 (51.4%)	0.909 (0.675–1.225)	
Pathologic Gleason grade group								
1 + 2	319 (60.2%)	102 (59.6%)	1.00	0.900	184 (58.2%)	237 (61.6%)	1.00	0.370
3 + 4 + 5	211 (39.8%)	69 (40.4%)	1.023 (0.720–1.454)		132 (41.8%)	148 (38.4%)	0.870 (0.643–1.719)	
Clinical T stage								
1 + 2	450 (84.9%)	153 (89.5%)	1.00	0.134	268 (84.8%)	335 (87.0%)	1.00	0.403
3 + 4	80 (15.1%)	18 (10.5%)	0.662 (0.384–1.139)		48 (15.2%)	50 (13.0%)	0.833 (0.544–1.278)	
Pathologic T stage								
2	286 (54.0%)	85 (49.7%)	1.00	0.332	158 (50.0%)	213 (55.3%)	1.00	0.160
3 + 4	244 (46.0%)	86 (50.3%)	1.186 (0.840–1.675)		158 (50.0%)	172 (44.7%)	0.808 (0.599–1.088)	
Pathologic N stage								
N0	485 (91.5%)	156 (91.2%)	1.00	0.909	289 (91.5%)	352 (91.4%)	1.00	0.990
N1	45 (8.5%)	15 (8.8%)	1.036 (0.562–1.910)		27 (8.5%)	33 (8.6%)	1.003 (0.590–1.708)	
Seminal vesicle invasion								
No	419 (79.1%)	133 (77.8%)	1.00	0.722	242 (76.6%)	310 (80.5%)	1.00	0.205
Yes	111 (20.9%)	38 (22.2%)	1.079 (0.711–1.636)		74 (23.4%)	75 (19.5%)	0.791 (0.551–1.137)	
Perineural invasion								
No	144 (27.2%)	42 (24.6%)	1.00	0.502	80 (25.3%)	106 (27.5%)	1.00	0.508
Yes	386 (72.8%)	129 (75.4%)	1.146 (0.770–1.705)		236 (74.7%)	279 (72.5%)	0.892 (0.636–1.251)	
Lymphovascular invasion								
No	453 (85.5%)	137 (80.1%)	1.00	0.095	265 (83.9%)	325 (84.4%)	1.00	0.841
Yes	77 (14.5%)	34 (19.9%)	1.460 (0.934–2.282)		51 (16.1%)	60 (15.6%)	0.959 (0.639–1.441)	
Biochemical recurrence								
No	362 (68.3%)	177 (68.4%)	1.00	0.977	213 (67.4%)	266 (69.1%)	1.00	0.633
Yes	168 (31.7%)	54 (31.6%)	0.995 (0.686–1.441)		103 (32.6%)	119 (30.9%)	0.925 (0.672–1.273)	
D'Amico classification								
Low risk/Intermediate risk	257 (48.5%)	91 (53.2%)	1.00	0.283	159 (50.3%)	189 (49.1%)	1.00	0.747
High risk	273 (51.5%)	80 (46.8%)	0.828 (0.586–1.169)		157 (49.7%)	196 (50.9%)	1.050 (0.780–1.414)	

*Note*: ORs with their 95% CIs were estimated by logistic regression models.

Abbreviation: PSA, prostate‐specific antigen.

**TABLE 4:** Odds ratios (ORs) and 95% confidence intervals (CIs) of the clinical status and *CHI3L1* rs880633 and rs10399805 genotypic frequencies in 701 patients with prostate cancer.

Variable	rs880633				rs10399805			
	TT (*N* = 305)	TC + CC (*N* = 396)	OR (95% CI)	*p‐*value	GG (*N* = 337)	GA + AA (*N* = 364)	OR (95% CI)	*p‐*value
PSA at diagnosis (ng/mL)								
≤10	145 (47.5%)	188 (47.5%)	1.00	0.986	158 (46.9%)	175 (48.1%)	1.00	0.752
>10	160 (52.5%)	208 (52.5%)	1.003 (0.744–1.352)		179 (53.1%)	189 (51.9%)	0.953 (0.709–1.283)	
Pathologic Gleason grade group								
1 + 2	188 (61.6%)	233 (58.8%)	1.00	0.453	198 (58.8%)	233 (61.3%)	1.00	0.498
3 + 4 + 5	117 (38.4%)	163 (41.2%)	1.124 (0.828–1.526)		139 (41.2%)	141 (38.7%)	0.901 (0.666–1.219)	
Clinical T stage								
1 + 2	268 (87.9%)	335 (84.6%)	1.00	0.215	286 (84.9%)	317 (87.1%)	1.00	0.397
3 + 4	37 (12.1%)	61 (15.4%)	1.319 (0.850–2.046)		51 (15.1%)	47 (12.9%)	0.831 (0.542–1.275)	
Pathologic T stage								
2	170 (55.7%)	201 (50.8%)	1.00	0.190	171 (50.7%)	200 (54.9%)	1.00	0.265
3 + 4	135 (44.3%)	195 (49.2%)	1.222 (0.905–1.649)		166 (49.3%)	164 (45.1%)	0.845 (0.628–1.137)	
Pathologic N stage								
N0	276 (90.5%)	365 (92.2%)	1.00	0.431	309 (91.7%)	332 (91.2%)	1.00	0.819
N1	29 (9.5%)	31 (7.8%)	0.808 (0.476–1.373)		28 (8.3%)	32 (8.8%)	1.064 (0.626–1.808)	
Seminal vesicle invasion								
No	247 (81.0%)	305 (77.0%)	1.00	0.204	262 (77.7%)	290 (79.7%)	1.00	0.534
Yes	58 (19.0%)	91 (23.0%)	1.271 (0.878–1.839)		75 (22.3%)	74 (20.3%)	0.891 (0.621–1.280)	
Perineural invasion								
No	79 (25.9%)	107 (27.0%)	1.00	0.739	85 (25.2%)	101 (27.7%)	1.00	0.449
Yes	226 (74.1%)	289 (73.0%)	0.944 (0.673–1.325)		252 (74.8%)	263 (72.3%)	0.878 (0.627–1.229)	
Lymphovascular invasion								
No	253 (83.0%)	337 (85.1%)	1.00	0.439	283 (84.0%)	307 (84.3%)	1.00	0.895
Yes	52 (17.0%)	59 (14.9%)	0.852 (0.567–1.280)		54 (16.0%)	57 (15.7%)	0.973 (0.649–1.460)	
Biochemical recurrence								
No	214 (70.2%)	265 (66.9%)	1.00	0.360	230 (68.2%)	249 (68.4%)	1.00	0.964
Yes	91 (29.8%)	131 (33.1%)	1.163 (0.842–1.605)		107 (31.8%)	115 (31.6%)	0.993 (0.722–1.365)	
D'Amico classification								
Low risk/Intermediate risk	159 (52.1%)	189 (47.7%)	1.00	0.248	168 (49.9%)	180 (49.5%)	1.00	0.915
High risk	146 (47.9%)	207 (52.3%)	1.193 (0.885–1.608)		169 (50.1%)	184 (50.5%)	1.016 (0.756–1.367)	

*Note*: ORs with their 95% CIs were estimated by logistic regression models.

Abbreviation: PSA, prostate‐specific antigen.

**TABLE 5:** Odds ratios (ORs) and 95% confidence intervals (CIs) of the clinical status and *CHI3L1* rs6691378 genotypic frequencies in 405 prostate cancer patients aged >65 years.

Variable	Genotypic frequencies			
rs6691378	GG (*N* = 174)	GA + AA (*N* = 231)	OR (95% CI)	*p‐*value
PSA at diagnosis (ng/mL)				
≤10	69 (39.7%)	104 (45.0%)	1.00	0.280
>10	105 (60.3%)	127 (55.0%)	0.802 (0.538–1.196)	
Pathologic Gleason grade group				
1 + 2	89 (51.1%)	142 (61.5%)	**1.00**	**0.038** *
3 + 4 + 5	85 (48.9%)	89 (38.5%)	**0.656 (0.441–0.977)**	
Clinical T stage				
1 + 2	136 (78.2%)	197 (85.3%)	1.00	0.064
3 + 4	38 (21.8%)	34 (14.7%)	0.618 (0.370–1.030)	
Pathologic T stage				
2	73 (42.0%)	128 (55.4%)	1.00	**0.007** *
3 + 4	101 (58.0%)	103 (44.6%)	**0.582 (0.391**–**0.865)**	
Pathologic N stage				
N0	154 (88.5%)	206 (89.2%)	1.00	0.831
N1	20 (11.5%)	25 (10.8%)	0.934 (0.501–1.744)	
Seminal vesicle invasion				
No	120 (69.0%)	188 (81.4%)	1.00	**0.004** *
Yes	54 (31.0%)	43 (18.6%)	**0.508 (0.320–0.806)**	
Perineural invasion				
No	36 (20.7%)	62 (26.8%)	1.00	0.153
Yes	138 (79.3%)	169 (73.2%)	0.711 (0.445–1.136)	
Lymphovascular invasion				
No	138 (79.3%)	197 (85.3%)	1.00	0.116
Yes	36 (20.7%)	34 (14.7%)	0.662 (0.395–1.109)	
Biochemical recurrence				
No	111 (63.8%)	162 (70.1%)	1.00	0.178
Yes	63 (36.2%)	69 (29.9%)	0.750 (0.494–1.140)	
D'Amico classification				
Low risk/Intermediate risk	67 (38.5%)	106 (45.9%)	1.00	0.137
High risk	107 (61.5%)	125 (54.1%)	0.738 (0.495–1.102)	

*Note*: ORs with their 95% CIs were estimated by logistic regression models.

Abbreviation: PSA, prostate‐specific antigen.

* *p* < 0.05 as statistically significant values in bold.

**TABLE 6:** Odds ratios (ORs) and 95% confidence intervals (CIs) of the clinical status and *CHI3L1* rs10399805 genotypic frequencies in 405 prostate cancer patients aged >65 years.

Variable	Genotypic frequencies			
rs10399805	GG (*N* = 186)	GA + AA (*N* = 219)	OR (95% CI)	*p‐*value
PSA at diagnosis (ng/mL)				
≤10	74 (39.8%)	99 (45.2%)	1.00	0.272
>10	112 (60.2%)	120 (54.8%)	0.801 (0.539–1.190)	
Pathologic Gleason grade group				
1 + 2	95 (51.1%)	136 (62.1%)	1.00	**0.026** *
3 + 4 + 5	91 (48.9%)	83 (37.9%)	**0.637 (0.429**–**0.947)**	
Clinical T stage				
1 + 2	147 (79.0%)	186 (84.9%)	1.00	0.122
3 + 4	39 (21.0%)	33 (15.1%)	0.669 (0.401–1.115)	
Pathologic T stage				
2	78 (41.9%)	123 (56.2%)	1.00	**0.004** *
3 + 4	108 (58.1%)	96 (43.8%)	**0.564 (0.380**–**0.837)**	
Pathologic N stage				
N0	166 (89.2%)	206 (88.6%)	1.00	0.832
N1	20 (10.8%)	25 (11.4%)	1.070 (0.573–1.995)	
Seminal vesicle invasion				
No	132 (71.0%)	176 (80.4%)	1.00	**0.027** *
Yes	54 (29.0%)	43 (19.6%)	**0.597 (0.377**–**0.946)**	
Perineural invasion				
No	36 (19.4%)	62 (28.3%)	1.00	**0.036** *
Yes	150 (80.6%)	157 (71.7%)	**0.608 (0.381**–**0.970)**	
Lymphovascular invasion				
No	149 (80.1%)	186 (84.9%)	1.00	0.201
Yes	37 (19.9%)	33 (15.1%)	0.714 (0.426–1.197)	
Biochemical recurrence				
No	121 (65.1%)	152 (69.4%)	1.00	0.352
Yes	65 (34.9%)	67 (30.6%)	0.821 (0.541–1.244)	
D'Amico classification				
Low risk/Intermediate risk	70 (37.6%)	103 (47.0%)	1.00	0.057
High risk	116 (62.4%)	116 (53.0%)	0.680 (0.456–1.012)	

*Note*: ORs with their 95% CIs were estimated by logistic regression models.

Abbreviation: PSA, prostate‐specific antigen.

* *p* < 0.05 as statistically significant values in bold.

### 3.4 Impacts of *CHI3L1* genetic polymorphisms on *CHI3L1* expression

**FIGURE 1:**
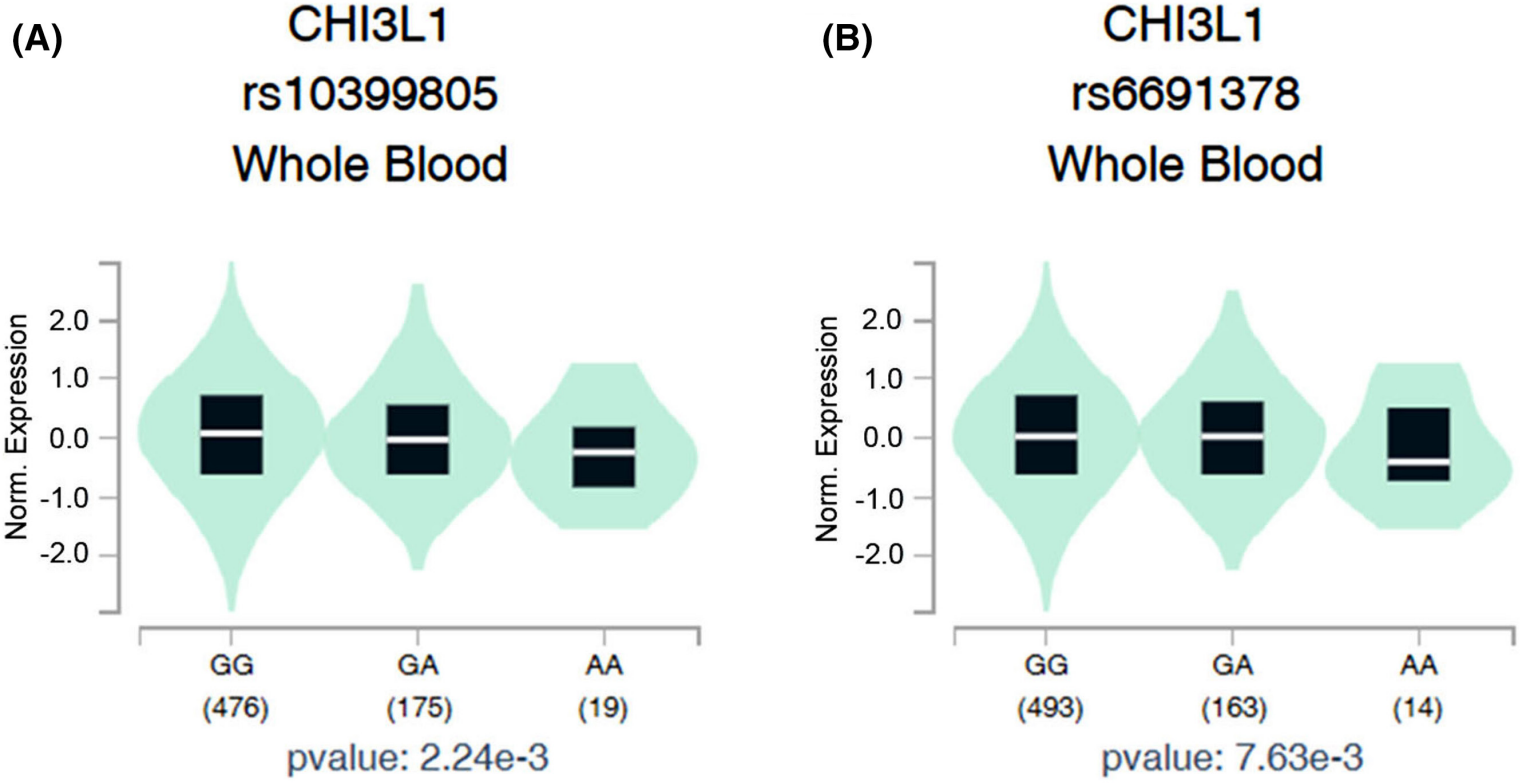
Impacts of chitinase 3‐like 1 (*CHI3L1*) rs6691378 and rs10399805 polymorphisms on CHI3L1 expression. The Genotype‐Tissue Expression (GTEx) Portal (https://www.gtexportal.org/home/) provided validated results regarding CHI3L1 expression based on different genotypes. Violin plots show that *CHI3L1* rs10399805 (A) and rs6691378 (B) mutations were associated with lower CHI3L1 expression levels in whole blood.

We subsequently assessed the association between *CHI3L1* polymorphisms and *CHI3L1* gene expression in whole blood tissues using samples from healthy individuals sourced from the Genotype‐Tissue Expression (GTEx) database. Individuals carrying the polymorphic A allele of rs10399805 (Figure 1A) and rs6691378 (Figure 1B) all exhibited lower *CHI3L1* expression compared with those with wild‐type homozygous genotypes.

### 3.5 Correlations of CHI3L1 expression levels with clinicopathologic features and prognoses of PCa patients

**FIGURE 2:**
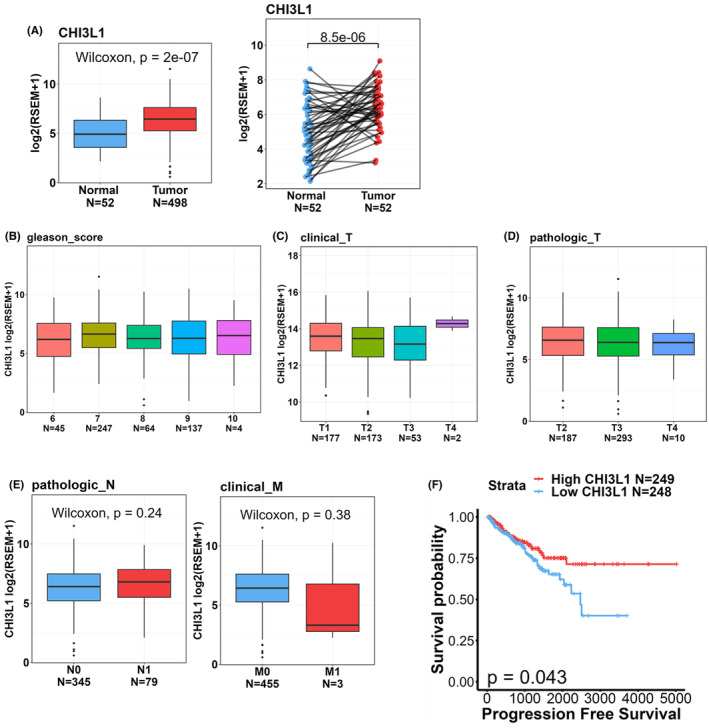
Clinical relevance of chitinase 3‐like 1 (CHI3L1) levels in prostate cancer (PCa) patients obtained from TCGA‐prostate adenocarcinoma (PRAD) dataset. (A) *CHI3L1* gene expression in unpaired (left panel) and paired (right panel) normal and tumour tissues derived from patients with PCa. (B–E) CHI3L1 expression levels in PCa from TCGA‐PRAD were compared according to the Gleason score (B), clinical T stage (C), pathological T stage (D), and lymph node (E, left panel) and distal metastasis (E, right panel). (F) Kaplan–Meier curves for progression‐free survival of patients with PCa, as categorized according to high or low CHI3L1 expression. The *P‐*value indicates a comparison between patients with CHI3L1^high^ and CHI3L1^low^ (database source: TCGA‐PRAD).

To perform a more comprehensive analysis of CHI3L1 expression levels in both normal and PCa tissues and to explore potential correlations of CHI3L1 levels with the progression and prognosis of PCa, we made use of TCGA‐PRAD dataset. Our examination of the dataset revealed that CHI3L1 expression levels were notably higher in tumour tissues compared with noncancerous tissues (Figure 2A, left panel) and to corresponding matched normal tissues (Figure 2A, right panel). Nevertheless, our investigation identified no significant correlations between elevated CHI3L1 expression levels and various clinicopathological features, including the Gleason score (Figure 2B), clinical stage (Figure 2C), pathological T stage (Figure 2D) and lymph node (Figure 2E, left panel) or distal metastasis (Figure 2E, right panel). Furthermore, when examining survival data, a Kaplan–Meier plot showed that PCa patients from TCGA‐PRAD dataset who exhibited CHI3L1^low^ tumours experienced shorter progression‐free survival (PFS) times compared with those with CHI3L1^high^ tumours (Figure 2F).

## 4 DISCUSSION

*CHI3L1/YKL40* genetic variants were demonstrated to impact its messenger (m)RNA expression and exhibit strong associations with various diseases, including asthma, Alzheimer's disease (AD), atopy and hypertension. For instance, Tsai et al. found that the rs10399931 GG genotype was linked to heightened serum YKL40 levels and increased severity of lung obstruction in asthma patients from southern Taiwan using steroids.^25^ Additionally, Dai et al. revealed that polymorphisms in the *CHI3L1* gene (such as rs4950928 and rs10399931) were connected with the AD risk and prognosis, potentially influencing CHI3L1's expression in plasma.^26^ Sohn et al. reported an association between the rs10399805 polymorphism in the promoter region of the *CHI3L1* gene and atopy. Individuals with the TT genotype exhibited a 2.5‐fold increase in *CHI3L1* mRNA expression in peripheral blood cells compared with those with the CC genotype.^27^ Moreover, the rs10399805, rs4950928 and rs2297839 genotypes in the *CHI3L1* gene were identified as stable biomarkers for predicting a hypertension risk.^28^ Over the past decade, significant attention has been directed towards exploring the potential role of CHI3L1 in the development of various human cancers. Several reports highlighted a substantial correlation between *CHI3L1* genetic variants and the risk, clinicopathologic progression and prognosis of various cancer types. For instance, the rs880633 polymorphism within the *CHI3L1* gene was identified as being correlated with the risk, progression and OS rate of patients afflicted with hepatocellular carcinoma (HCC). Notably, patients carrying the CC genotype of rs880633 displayed elevated serum CHI3L1 levels, whereas individuals with the TT genotype exhibited the lowest serum CHI3L1 levels.^29^ Furthermore, two *CHI3L1* SNPs, namely rs6691378 and rs10399805, were associated with the development of cervical precancerous lesions and invasive cancer.^22^ Despite these findings, clinical implications of *CHI3L1* SNPs in PCa remain largely unexplored. These SNPs potentially lead to expression and functional alterations of CHI3L1, which could influence PCa progression. Herein, we found for the first time that genetic variants in *CHI3L1* play pivotal roles in shaping clinicopathological characteristics of PCa within a Taiwanese population.

PCa is mainly a disease of seniors aged 60–70 years.^1^ This study presents findings that individuals aged over 65 years, possessing the mutated base A variant of *CHI3L1* rs6691378, exhibited a notably reduced risk of developing high Gleason grade (3–5), advanced T stage (3 or 4) and seminal vesicle invasion under a dominant genetic model (GA + AA). Furthermore, our investigation revealed that elderly PCa patients carrying the GA or AA genotype of rs10399805 similarly demonstrated significantly diminished risks of developing a high Gleason grade, advanced T stage, and seminal vesicle and perineural invasion. These results were similar to observations of our previous study, which indicated that oral cancer patients who had at least one mutant A allele of *CHI3L1* rs6691378 and rs10399805 had a significantly lower frequency of developing lymph node metastasis.^21^ These insights shed light on the potential influence of specific *CHI3L1* genetic variants on disease progression and clinical characteristics of PCa among older individuals.

Both rs6691378 and rs10399805 SNPs are situated within the promoter regions of the *CHI3L1* gene. Rehli et al. identified various transcription factor‐binding sites in the *CHI3L1* gene promoter, including C/EBP‐ and AML‐1‐binding sites within the region spanning positions −234 to −252 relative to the ATG start site of transcription.^30^ Notably, rs10399805 resides at position −247 and was reported to influence CHI3L1's transcriptional activity. For instance, Sohn et al. conducted an in vitro promoter assay using THP‐1 cells which revealed that a C/G to T/A conversion at the rs10399805 SNP led to an increase in reporter gene expression. Furthermore, the −247T allele exhibited heightened affinity for C/EBP, as determined by an electrophoretic mobility shift assay. In an in vivo context, individuals with the TT/AA genotype exhibited elevated *CHI3L1* mRNA expression in peripheral blood cells compared with those carrying the CC/GG genotype.^27^ Those findings suggested that the T/A allele of rs10399805 may contribute to promotion of CHI3L1 expression. Indeed, the T/A allele of rs10399805 was associated with elevated serum CHI3L1 levels in patients with atopy^27^ and hypertension.^28^ However, this correlation was observed not to be significant in patients with coronary artery disease.^31^ In contrast, data extracted from the GTEx database showed decreased *CHI3L1* expression in whole blood tissues among individuals carrying the polymorphic A allele of rs10399805. This discrepancy could potentially be attributed to variations in study populations or disease‐related risk profiles. In addition to rs10399805, there is currently no reported correlation between the rs6691378 SNP and serum CHI3L1 levels in any disorder. To address this gap, we conducted preliminary assessments of the association between *CHI3L1* rs6691378 polymorphisms and *CHI3L1* gene expression from the GTEx database, where we observed decreased *CHI3L1* expression in whole blood tissues among individuals carrying the polymorphic A allele of *CHI3L1* rs6691378. Nevertheless, correlations of rs10399805 and rs6691378 SNPs with serum CHI3L1 levels in patients with PCa warrant further investigation in future work.

With our current understanding, CHI3L1 was previously observed to be upregulated in various types of solid tumours, including breast cancer,^32^ colon cancer,^33^ ovarian cancer,^34^ glioblastoma multiforme (GBM)^35^ and PCa.^17^ Elevated CHI3L1 levels were associated with unfavourable prognoses and reduced survival rates in breast, colon and ovarian cancer patients.^32^, ^33^, ^34^ In line with these previous studies, our findings indicated that *CHI3L1* transcripts in PCa were significantly higher compared with those in noncancerous tissues or matched normal tissues from TCGA‐PRAD dataset. Contrary to the common trend of poor prognostic impacts associated with high CHI3L1 expression, our results surprisingly revealed that elevated *CHI3L1* expression was correlated with a longer PFS in PCa patients. This observation suggests that CHI3L1 might play a tumour‐suppressive role in the context of PCa. Notably, recent research highlighted the dual nature of CHI3L1 in cancer. For instance, in GBM stem‐like cells (GSCs) with methylated O6‐methylguanine‐DNA methyltransferase promoter (MGMT‐m), *CHI3L1* functioned as a tumour suppressor gene, sensitizing GSCs' response to temozolomide (TMZ) by activating DNA damage responses (DDRs). In contrast, in MGMT promoter‐unmethylated (MGMT‐um) GSCs, it promoted tumorigenesis and contributed to TMZ resistance by inhibiting DDRs.^36^ This indicates that the methylation status of the MGMT promoter might influence CHI3L1's function in human cancers.

Our current study still has some limitations that need to be considered. First, our study only recruited a Taiwanese population. Including other ethnic populations in future studies will allow for comparisons and validation of the findings across different racial groups. Additionally, owing to the relatively small sample size, the frequencies of some homozygous variants were low in subgroups and therefore may limit the statistical power and precision of the results. Therefore, conducting larger independent cohorts from different medical centres can provide more robust and reliable findings regarding the impact of CHI3L1 SNPs on the risk and development of PCa. Moreover, our current study only indicated the impacts of CHI3L1 rs10399805 and rs6691378 SNPs on CHI3L1 gene expression in whole blood tissues among healthy individuals based on the GTEx database. To further validate the influence of CHI3L1 SNPs on CHI3L1 expression in PCa patients, mRNA and DNA should be collected simultaneously from the same samples from PCa patients in future work.

In summary, our study identified distinct allelic effects of CHI3L1 SNPs (rs10399805 and rs6691378) within a Taiwanese population, that impact the clinicopathologic development of PCa. Furthermore, we uncovered a prognostic role for CHI3L1 in PCa using clinical samples. Our results suggest that the promoter SNPs, rs10399805 and rs6691378, might influence *CHI3L1* gene expression, subsequently modulating PCa progression. These genetic variants could potentially serve as critical markers to predict the aggressiveness and prognosis of PCa.

## AUTHOR CONTRIBUTIONS

**Yu‐Ching Wen:** Conceptualization (equal); data curation (equal); funding acquisition (equal); writing – original draft (equal). **Chia‐Yen Lin:** Data curation (equal); resources (equal). **Kuo‐Hao Ho:** Data curation (equal); software (equal). **Yung‐Wei Lin:** Conceptualization (equal); methodology (equal). **Chi‐Hao Hsiao:** Conceptualization (equal). **Shian‐Shiang Wang:** Data curation (equal); resources (equal). **Lun‐Ching Chang:** Software (equal). **Shun‐Fa Yang:** Conceptualization (equal); methodology (equal); writing – original draft (equal). **Ming‐Hsien Chien:** Conceptualization (equal); funding acquisition (equal); software (equal); writing – original draft (equal); writing – review and editing (equal).

## CONFLICT OF INTEREST STATEMENT

The authors declare no conflicts of interest related to this study.

## Data Availability

The data used to support the findings of this study are available from the corresponding author upon reasonable request.
